# Point-of-care Ultrasound in the Evaluation of Mitral Valve Regurgitation and Mitral Annular Calcification

**DOI:** 10.5811/cpcem.2020.7.48117

**Published:** 2020-09-17

**Authors:** Benjamen Schoenberg, Marwan Alkhattabi, Shadi Lahham

**Affiliations:** * University of California, Riverside, Riverside Community Hospital, Department of Emergency Medicine, Riverside, California; † University of California, Irvine, Department of Emergency Medicine, Orange, California

**Keywords:** Mitral valve calcification, mitral valve regurgitation, cardiac ultrasound, point-of-care ultrasound

## Abstract

**Case Presentation:**

A 77-year-old female presented to the emergency department (ED) with chest pain. Cardiac point-of-care ultrasound (POCUS) was performed and demonstrated a hyperechoic structure on the posterior leaflet of the mitral valve. Admission to cardiology and echocardiogram revealed moderately decreased mobility of the posterior leaflet, mitral annular calcification, and severe mitral regurgitation.

**Discussion:**

These findings highlight the role of POCUS in identifying mitral valve pathology in the ED, ultimately leading to appropriate disposition and management. Mitral annular calcification can lead to significant manifestations including mitral stenosis or regurgitation, and advanced cases have been associated with an increased risk of infective endocarditis, thrombosis, and arrhythmia.

## CASE PRESENTATION

A 77-year-old female with a history of atrial fibrillation on apixaban, non-ST-elevation myocardial infarction (NSTEMI), congestive heart failure with preserved ejection fraction, and hypertension presented to the emergency department (ED) with chest pain. The patient described non-exertional, sudden-onset and severe left-sided chest pain radiating to her left shoulder. Review of systems was positive for shortness of breath, nausea, and diaphoresis. Vital signs were notable for an elevated heart rate and respiratory rate. Physical exam revealed jugular venous distension, irregular rate and rhythm, and 2+ lower extremity edema. Electrocardiogram revealed atrial fibrillation, and labs were significant for an elevated high-sensitivity troponin.

Point-of-care ultrasound (POCUS) in parasternal long view of the heart revealed left atrial enlargement, left ventricular hypertrophy, mitral valve regurgitation, and a hyperechoic structure on the posterior leaflet of the mitral valve representing moderate mitral annular calcification (MAC) with posterior shadowing (Image and Video). The patient was admitted to cardiology for atrial fibrillation with NSTEMI and further management.

## DISCUSSION

These findings highlight the role of POCUS in identifying mitral valve pathology in the ED, ultimately leading to appropriate disposition and management. Mitral regurgitation (MR) is the leading cause of valvular heart disease in the United States with a prevalence of 10% in adults older than 75 years.^1^ Severe MR can present with a holosystolic heart murmur, dyspnea on exertion, lightheadedness, cough, palpitations, pulmonary congestion, and edema.^1^

MAC is a progressive, degenerative process that is a result of calcification of the fibrous mitral annulus.^2^ MAC can lead to significant manifestations including mitral stenosis or regurgitation (such as in this patient); and advanced cases have been associated with an increased risk of infective endocarditis, thrombosis, and arrhythmia.^3^ Risk factors for MAC include advanced age, diabetes mellitus, hyperlipidemia, chest radiation, chronic kidney disease, and hypertension.^4^

## LIMITATIONS

A color Doppler image or video to evaluate MR was not saved in the electronic health record.

CPC-EM Capsule

What do we already know about this clinical entity?

*Mitral regurgitation is the leading cause of valvular heart disease in the United States*.

What is the major impact of the image(s)?

*Mitral annular calcification can present as either an incidental finding on ultrasound or as a significant contributor in patients with mitral regurgitation*.

How might this improve emergency medicine practice?

*Point-of-care ultrasound is an important tool to evaluate patients presenting with chest pain and possible valvular pathology in the emergency department*.

## Supplementary Information

Video Cardiac point-of-care ultrasound revealing a hyperechoic structure on the posterior leaflet of the mitral valve representing calcification (black arrow) and left atrial enlargement (yellow star).

## Figures and Tables

**Image:**
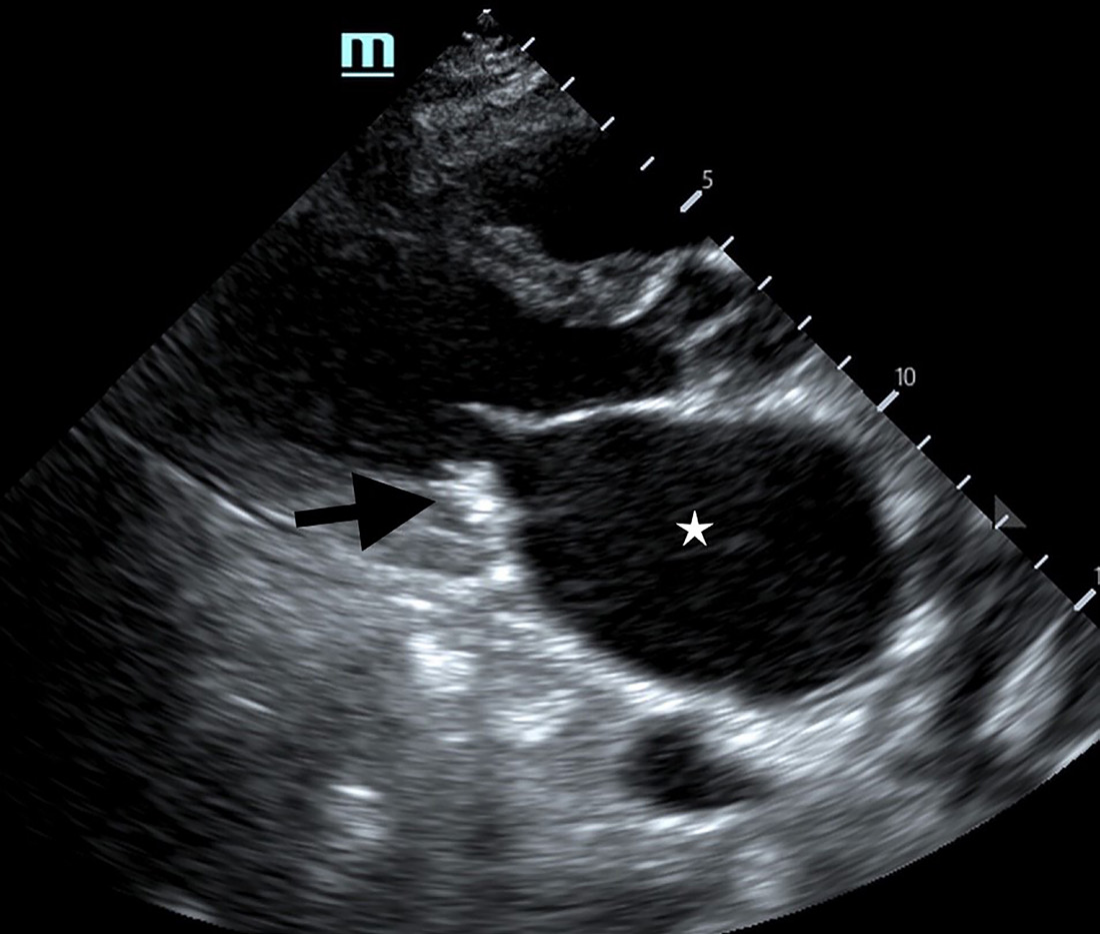
Point-of-care ultrasound in parasternal long view of the heart revealing a hyperechoic structure on the posterior leaflet of the mitral valve representing calcification (black arrow) and left atrial enlargement (white star).
